# Enhanced Interfacial Properties of Carbon Fiber/Maleic Anhydride-Grafted Polypropylene Composites via Two-Step Surface Treatment: Electrochemical Oxidation and Silane Treatment

**DOI:** 10.3390/polym15183784

**Published:** 2023-09-16

**Authors:** Dong-Kyu Kim, Woong Han, Kwan-Woo Kim, Byung-Joo Kim

**Affiliations:** 1 Industrialization Division, Korea Carbon Industry Promotion Agency, Jeonju 54852, Republic of Korea; kdg9141@kcarbon.or.kr (D.-K.K.); shareyi@kcarbon.or.kr (W.H.); 2 Department of Carbon Materials and Fiber Engineering, Jeonbuk University, Jeonju 54896, Republic of Korea; 3 Department of Advanced Materials and Chemical Engineering, Jeonju University, Jeonju 55069, Republic of Korea

**Keywords:** carbon fiber, silane treatment, electrochemical oxidation, composites, interfacial shear strength

## Abstract

The interfacial adhesion between carbon fibers (CFs) and a thermoplastic matrix is an important aspect that should be improved in manufacturing CF-reinforced thermoplastics with high strength and rigidity. In this study, the effects of a two-step surface treatment comprising electrochemical oxidation and silane treatment of the CF surface on the mechanical properties of CF/maleic anhydride-grafted polypropylene (MAPP) composites were confirmed. The surface characteristics of the treated CFs were analyzed via scanning electron microscopy, atomic force microscopy, Fourier transform infrared spectroscopy, and X-ray photoelectron spectroscopy. The tensile testing of a single CF and interfacial adhesion of the samples before and after the surface treatment were analyzed using a single-fiber testing machine and a universal testing machine. After the silane treatment, the roughness of the CF surface increased due to the formation of a siloxane network. In addition, the interfacial shear strength increased by ∼450% compared to that of the untreated CFs due to the covalent bond between the -NH_2_ end group of siloxane and MAPP. This two-step surface treatment, which can be performed continuously, is considered an effective method for improving the mechanical interface strength between the CF and polymer matrix.

## 1. Introduction

Carbon fibers are highly functional materials with advantageous characteristics, such as high strength, high elasticity, heat resistance, and light weight, and they are widely used as an ideal reinforcement for polymer matrix composites in various applications [1]. In general, carbon fibers are divided into polyacrylonitrile (PAN) [2], petroleum-based pitch [3], and cellulose (rayon) types [4] depending on the precursor. Among these, PAN-based carbon fibers with excellent mechanical properties are often used. PAN-based carbon fibers are used as a structural material for aerospace, defense, automobiles, and various high-performance carbon-fiber-reinforced plastics (CFRPs) owing to their superior tensile strength, modulus of elasticity, and chemical resistance compared to other industrial fibers [5,6,7]. In particular, with the development of new types of ecofriendly energy, such as high-performance batteries and fuel cells, new types of air transport devices, such as personal air vehicles and drones, are being developed for various purposes. The development of this new type of ecofriendly energy is actively shifting towards ecofriendly vehicles to address emission problems, such as volatile organic compounds (e.g., SO_X_ and NO_X_), which are the biggest problems of internal combustion engine vehicles and one of the main causes of environmental pollution, and CO_2_, which causes global warming [8,9]. Among such electric vehicles, purpose-built vehicles, which are representative ecofriendly vehicles that use batteries and driving systems for various purposes, are in the spotlight [10].

These new types of transportation have a common requirement of light weight, which is essential for energy efficiency. As such, lighter materials with higher strength than that of existing materials are required. Traditional materials based on steel and metal alloys have high strength and stiffness, but have the disadvantage in terms of weight reduction due to their high density. Thus, there is an emerging use of composites that can satisfy high strength and weight reduction. A composite is a material that combines two or more materials and maintains the characteristic of its components. A representative composite is fiber-reinforced plastics, in which a fiber-type reinforcing material to increase strength is combined with a light polymer plastic base material with low density. Fiber-reinforced plastics are classified into glass fiber-reinforced plastics [11], CFRPs [12], and aramid-reinforced plastics [13]. Among these, CFRPs with low density, high strength, and high rigidity are excellent alternatives to steel.

CFRPs are largely divided into carbon-fiber-reinforced thermosetting plastics (CFRSPs) and carbon-fiber-reinforced thermoplastics (CFRTPs). Compared with CFRSPs, CFRTPs can be used to produce lightweight metal replacement parts in engineering applications owing to their flexible manufacturing process, superior machinability, weldability, and recyclability [14]. The representative thermoplastic resins used in CFRTPs include polypropylene (PP), polyamide, and polycarbonate.

While CFRTPs have several advantages, most commercially produced carbon fibers are surface treated and sized for carbon-fiber-reinforced thermosetting composites, resulting in weak interfacial adhesion to the carbon fiber surface when combined with thermoplastic resins [15,16]. Improving the interfacial adhesion between carbon fibers and thermoplastics is important because a weak interfacial adhesion induces a low shear force and leads to the premature failure of composites. In particular, it is necessary to improve the interfacial adhesion with the thermoplastic matrix by modifying the inert carbon fiber surface to manufacture CFRTPs with high strength and rigidity [17,18,19]. Methods for modifying carbon fiber surfaces include gas-phase oxidation [20], liquid-phase oxidation [21], plasma oxidation [22], electrochemical oxidation [23], surface coating [24], and thermal treatment [25]. Among various surface treatments, electrochemical oxidation is preferred for commercial use due to the easy treatment process and increased polarity because it allows the introduction of oxygen functional groups and imparts roughness to the carbon fiber surface [26]. However, for a thermoplastic resin with an inert molecular structure, it is difficult to significantly improve the interfacial adhesion with carbon fibers via electrochemical oxidation. In addition, chemical oxidation and etching during treatment damage the carbon fiber, thereby deteriorating the mechanical properties [27].

When a silane coupling agent [28] is introduced, numerous functional groups that can react with the surface of the oxidized carbon fibers are formed. Silane coupling agents have a chemical structure of R_(4−*n*)_-Si-(R’X)*_n_* (*n* = 1, 2), where R is the alkoxy, X is the organofunctionality, and R’ is an alkyl bridge connecting the Si atom and organofunctionality. In the past decades, studies on silane treatment of various reinforcing materials, such as glass, carbon, and natural fibers, have been conducted with most of them using trialkoxysilanes. The organofunctionality of silanes interacts with the matrix depending on the polymer compatibility. The nonreactive alkyl groups of silanes increase compatibility owing to their similar polarity with nonpolar matrices. However, reactive organofunctionality can be physically compatible and covalently bound to the matrix. The organofunctionalities of silane are generally amino, mercapto, glycidoxy, vinyl, or methacryloxy groups. Among them, γ-aminopropyltriethoxysilane (APTS) has been frequently reported as a coupling agent between carbon fibers and a matrix [29]. The introduction of these silane coupling agents can effectively impart wettability to the surface of the carbon fibers and improve their compatibility with the matrix. In addition, they can supplement the physical properties of carbon fibers with reduced tensile strength due to the surface treatment [30,31,32]. Furthermore, chemical crosslinking, such as through covalent bonds, between the functional groups and matrix on the surface of silane-treated carbon fibers can occur, improving the interfacial adhesion of CFRTPs [33,34,35].

In this study, surface treatment was performed through silane treatment after the electrochemical oxidation of carbon fibers, and a CFRTP was fabricated using maleic-anhydride-grafted PP (MAPP) as a matrix to investigate the effect of surface treatment on interfacial adhesion.

## 2. Materials and Methods

### 2.1. Materials

In this study, PAN-based unsized carbon fibers (TZ-607, Taekwang Co., Seoul, Republic of Korea) were used, and the thermoplastic resin was MAPP (PH-200, MFI > 100 g/10 min, density = 0.36 g/cm^3^, MA graft ratio > 1 wt.%, Lotte Chem. Co., Seoul, Republic of Korea) was used as the matrix. The electrolyte used for electrochemical oxidation treatment was ammonium bicarbonate (NH_4_HCO_3_, Daejung Chem. Co., Siheung, Republic of Korea) and (3-Aminopropyl)triethoxysilane (APTS, Sigma-Aldrich, Burlington, MA, USA). Ethanol (Sigma-Aldrich, Burlington, MA, USA) and deionized water were used for the silane treatment.

### 2.2. Carbon Fiber Surface Treatment

The surface of the carbon fibers was first oxidized via electrochemical oxidation. Subsequently, the carbon fibers were immersed in 0.2 mol/L of ammonium bicarbonate solution. The carbon fibers and graphite plates were connected to the anode and cathode, respectively, and treated for 100 s at a current density of 1 A/m^2^. The oxidized carbon fibers were washed with distilled water and dried in an oven at 100 °C for 1 h. Silane treatment was then performed on the surface-oxidized carbon fibers. After the addition of ethanol and distilled water (19:10 vol.%) to a beaker, the mixture was stirred at 60 °C for 30 min with 25 mL of acetic acid. Subsequently, 1, 3, and 5 wt.% silane coupling agents were mixed with the aqueous solution. After further stirring at 60 °C for 30 min, the mixture was transferred to a treatment tank. Finally, silane treatment was performed by immersing the electrochemically oxidized carbon fibers in an aqueous solution of each silane coupling agent for 10 min. The silane-treated carbon fibers were dried in an oven at 80 °C for 24 h to prepare the samples. The samples were labelled according to their treatment conditions; the details are provided in Table 1.

### 2.3. Single-Carbon-Fiber Microdroplet Test

**Figure 1:**
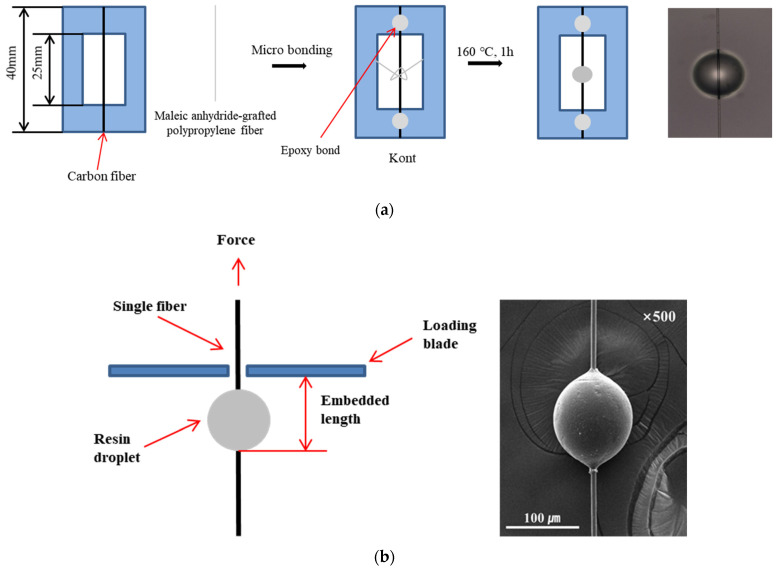
Schematic of the microbond test: (**a**) sample preparation and (**b**) debonding process.

Figure 1 shows the method for measuring the interfacial adhesion between the electrochemical-oxidation- and silane-treated carbon fibers and MAPP. First, a single carbon fiber was placed at the center of the paper frame and bonded with epoxy resin. The MAPP fiber was knotted in the middle of the carbon fiber placed at the center of the paper frame, melted at 160 °C for 1 h, and cooled to room temperature (25 °C) to produce a perfect sphere as the microdroplet test sample.

The interfacial shear strength (IFSS) of the fabricated samples was measured through the microdroplet test and calculated as follows:

(1) $$IFSS=\frac{F}{\pi DL}$$

where *F* is the peak pullout force (N), *D* is the fiber diameter (μm), and *L* is the embedded fiber length (μm) in the matrix. The results of more than 10 successful measurements were averaged.

### 2.4. Characterization

Scanning electron microscopy (SEM; AIS2000C, Seron Tech. Inc., Anseong, Republic of Korea) was used to examine the surface morphology before and after the surface treatment of the carbon fibers. Each sample was placed in a sample holder and coated with osmium to obtain clear images. All images were obtained at an acceleration voltage of 25 kV at 1.0 × 10^−5^ torr.

The surface of the carbon fibers was observed through atomic force microscopy (AFM; Park Systems Co., Suwon, Republic of Korea). The surface roughness was measured in the tapping mode. The scanning rate was 0.2 Hz, and the scanning scope was set to 5 × 5 µm.

The functional groups on the surface-treated carbon fibers were confirmed and analyzed by Fourier transform infrared (FTIR) spectroscopy (Nicolet^TM^ iS^TM^ 10, ThermoFisher Scientific, Waltham, MA, USA) at the wavenumber range from 4000 to 500 cm^−1^. The FTIR samples were prepared as discs by grinding the carbon fibers and potassium bromide (Sigma-Aldrich, Saint Louis, MO, USA) together and applying a clamp force of 7 tons for 2 min using a hydraulic press (CrushIR, PIKE Technologies, Madison, WI, USA).

The chemical components and relative contents of the functional groups on the carbon fiber surface were analyzed through X-ray photoelectron spectroscopy (XPS; PHI 5000 Versa Probe II, ULVAC-PHI, Chigasaki, Japan). Unless otherwise specified, the X-ray anode was operated at >5 W, and the voltage was maintained at 5.0 kV. The energy resolution was fixed at 0.50 eV to ensure sufficient sensitivity. The base pressure of the analyzer chamber was ~5 × 10^−8^ Pa. Both the full-scan (0–1200 eV) and narrow spectra were recorded with extremely high resolutions for individual elements. The binding energies were calibrated with respect to the adventitious carbon peak (C_1s_: 284.6 eV). The high-resolution C_1s_, O_1s_, Si_2p_, and N_1s_ peaks of the samples were deconvoluted using a Shirley-type baseline and iterative least-squared optimization algorithm. Furthermore, a curve-fitting procedure was carried out using a nonlinear least-square curve-fitting program with a Gaussian–Lorentzian production function.

The tensile properties of single carbon fibers were measured using a Favigraph semiautomatic device (Textechno Company, Mönchengladbach, Germany). The gauge length of the fiber was 20 mm, and the draw-off clamp speed was set to 1 mm/min. The filament was suspended between the grips of the testing machine. Load was applied to the carbon fiber until failure. The force–displacement curve was recorded. The microdroplet test for a single fiber was performed using a universal testing machine (UTM; Lloyd, UK) at a constant speed of 0.1 mm/min.

## 3. Results

### 3.1. Surface Morphology and Chemical Structural Analysis

**Figure 2:**
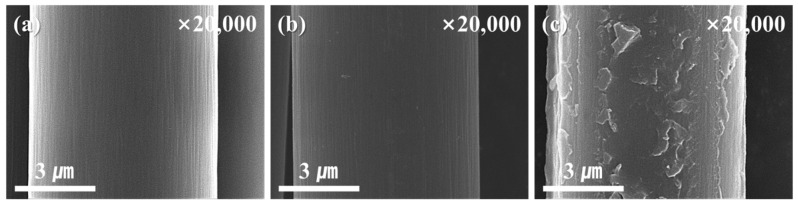
Scanning electron microscopy images of the surface of the untreated, electrochemical-oxidation-treated, and electrochemical-oxidation/silane-treated carbon fiber; (**a**) AS-CF, (**b**) EO-CF, and (**c**) EOS3-CF.

The SEM images shown in Figure 2 confirm the morphological changes of the carbon fibers according to the surface treatment. The AS-CF sample exhibits grooves along the fiber length direction, which became shallow after electrochemical oxidation. It is assumed that the deep grooves formed along the fiber length direction via the electrochemical oxidation treatment became shallow owing to the surface etching. According to previous studies, etching proceeds with electrochemical oxidation, thereby changing the diameter and decreasing the strength of the carbon fiber [23]. In this study, as the carbon fiber diameter did not change significantly after electrochemical oxidation, it can be inferred that etching did not proceed excessively. For the electrochemical-oxidation-treated and silane-treated carbon fibers, a partially overtreated silane layer was observed. The resulting shape can supplement surface cracks by increasing the interfacial adhesion owing to the increase in the roughness of the carbon fiber surface and filling the grooves formed along the length direction.

**Figure 3:**
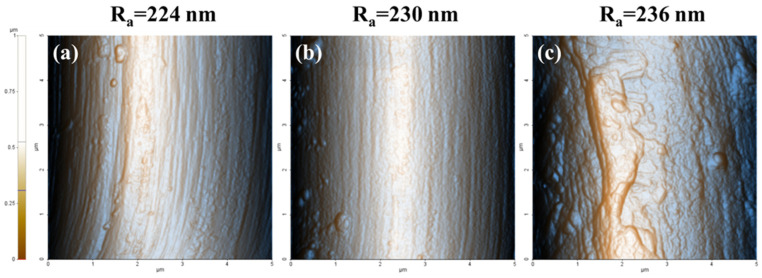
Atomic force microscopy images of the surface of the untreated, electrochemical-oxidation-treated, and electrochemical-oxidation/silane-treated carbon fiber; (**a**) AS-CF, (**b**) EO-CF, and (**c**) EOS3-CF.

Figure 3 shows the AFM images of the carbon fibers according to the surface treatment conditions. For AS-CF, wide and narrow grooves are observed on the fiber surface, and the average roughness (R_a_) was measured to be ~224 nm. The R_a_ values of EO-CF and EOS1-CF slightly increase to approximately 230 and 236 nm, respectively. This indicates that the roughness increased because of the formation of small grooves on the carbon fiber surface due to etching during electrochemical oxidation, thereby slightly increasing R_a_. After silane treatment, several silane layers formed on the surface of the carbon fiber, and R_a_ increased, which can improve the bonding strength with the thermoplastic resin.

**Figure 4:**
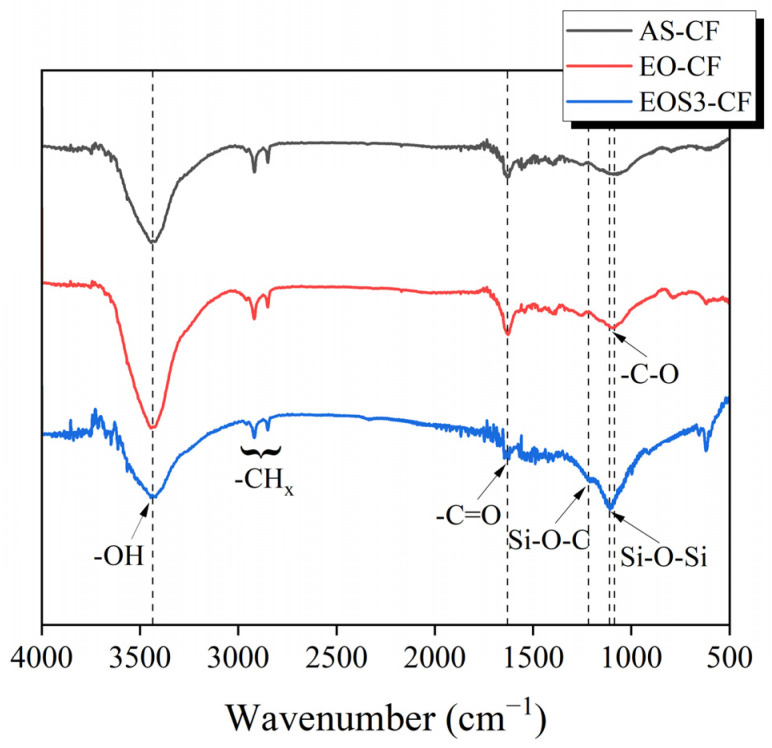
Fourier transform infrared spectra of the untreated, electrochemical-oxidation-treated, and electrochemical-oxidation/silane-treated carbon fibers.

**Figure 5:**
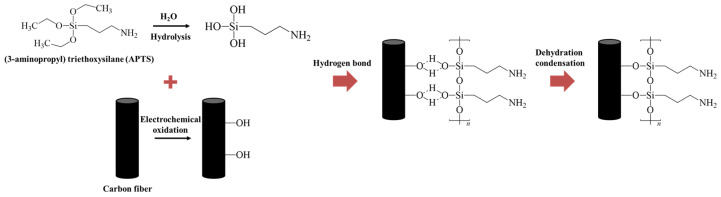
Chemical reaction of silane on the oxidized carbon fiber surfaces.

Figure 4 shows the FTIR spectra, revealing the changes in the functional group according to the surface treatment. In the FTIR spectra of AS-CF, stretching peaks of hydroxyl groups (-OH), carbonyl groups (-C=O), and carboxyl groups (-C-O), which are oxygen functional groups, are identified at 3440, 1640, and 1250–1050 cm^−1^ [27]. After electrochemical oxidation, the intensity of the oxygen functional group peaks increased, indicating that oxygen was introduced to the carbon fiber surface during electrochemical oxidation. In the silane-treated samples, the contents of the oxygen functional groups were lower than those of EO-CF. Moreover, new Si-O_X_ peaks were observed between 1350 and 1100 cm^−1^ in the FTIR spectra of the silane-treated samples [27]. It is believed that the oxygen functional groups formed on the surface of the electrochemical-oxidation-treated carbon fibers reacted with the -OH functional group of hydrolyzed silane, reducing the content of the oxygen functional group and forming the Si-O_X_ group owing to the formation of the siloxane network. This mechanism is illustrated in Figure 5. The silane coupling agent with an R_(4−*n*)_-Si-(R’X)*_n_* (*n* = 1,2) structure was hydrolyzed via treatment with water and alcohol to produce silanol. The silanol produced by the hydrolysis reaction initiated a condensation reaction with the neighboring silanol to form an oligomer. Subsequently, the hydrogen bonds with -OH groups formed on the surface of the electrochemical-oxidation-treated carbon fiber were condensed in the drying step, thereby forming a siloxane network with Si-O bonds (-Si-O-Si-). The siloxane network formed on the carbon fiber surface can affect the improvement of the interfacial adhesion through the covalent bond between the end group -NH_2_ and MAPP.

**Figure 6:**
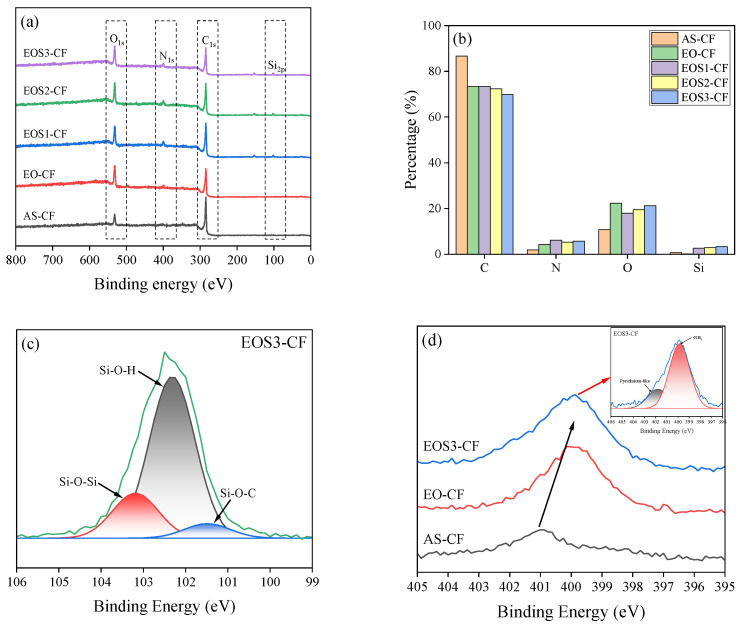
X-ray photoelectron spectra of the carbon fiber samples; (**a**) wide-scan survey, (**b**) surface element concentration of the carbon fiber samples, (**c**) fitting curve of the Si_2p_ peaks of EOS3-CF, (**d**) N_1s_ spectra of the carbon fiber samples subjected to different treatments.

XPS was conducted to investigate the chemical composition of the surfaces of the carbon fibers subjected to the proposed surface treatment process; the spectra are shown in Figure 6. The surface of AS-CF is mainly composed of carbon, nitrogen, and oxygen. In EO-CF, the oxygen content increased from 10.71% to 22.29%, the nitrogen content increased from 1.91% to 4.28%, and the carbon content decreased from 86.72% to 73.30%. This result shows that electrochemical oxidation treatment oxidized the carbon fiber surface, promoting the production of several oxygen-containing groups and activating the carbon fiber surface. The nitrogen and silicon contents of the silane-treated samples increased after silane treatment, indicating the formation of a siloxane network via the hydrogen bonding of the -OH functional and silanol groups generated on the carbon fiber surface after electrochemical oxidation.

High-resolution XPS Si_2ps_ and N_1_ spectra are shown in Figure 6c,d, respectively. The deconvolution of the N_1s_ and Si_2p_ spectra yielded several peaks, representing Si-O-Si (101.5 eV), Si-O-H (102.3 eV), Si-O-C (103.1 eV), -NH_2_ (399.8 eV), and pyridinium-like structures (401.7 eV) [27,36]. In Figure 6c, the Si_2p_ spectra confirm the formation of silane functional groups, such as Si-O-C, Si-O-H, and Si-O-Si, due to the combination of the -OH functional and silanol groups on the carbon fiber surface. In addition, as shown in Figure 6d, the N_1s_ spectra confirmed the intensity increase and shift after silane treatment. It is believed that the -NH_2_ functional group was formed on the carbon fiber surface under the influence of the -NH_2_ functional group at the end of the silanol group when the aqueous solution was used during electrochemical oxidation and silane treatment. Thus, the proposed surface treatment plays an important role in improving the binding force between the carbon fibers and matrix by introducing oxygen- and nitrogen-containing functional groups.

### 3.2. Mechanical Property Analysis

**Figure 7:**
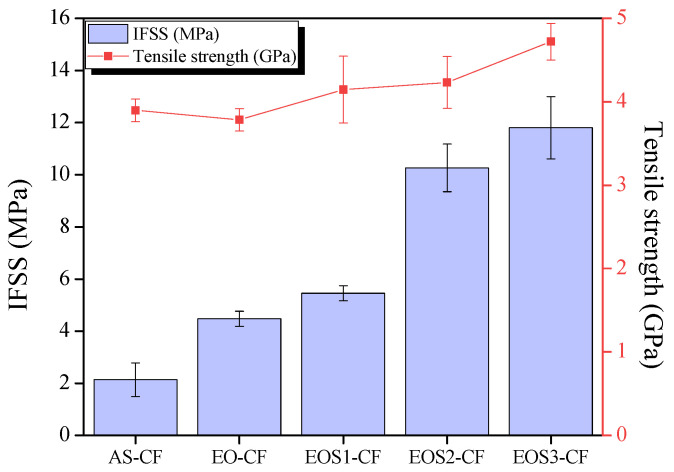
Interfacial shear strength and tensile test results of the untreated and surface-treated carbon fiber samples subjected to different conditions.

Figure 7 shows the tensile properties of single carbon fibers and the IFSS of untreated and surface-treated carbon fibers. The tensile strength of EO-CF is lower than that of AS-CF because chemical etching during electrochemical oxidation damaged the surface structure of the carbon fiber [23]. However, after silane treatment, the tensile strength of EOS3-CF increased by approximately 21% compared to that of AS-CF. The silane layer formed on the carbon fiber surface covered the surface crack when a tensile load was applied, thereby increasing the tensile strength [15,27].

**Figure 8:**
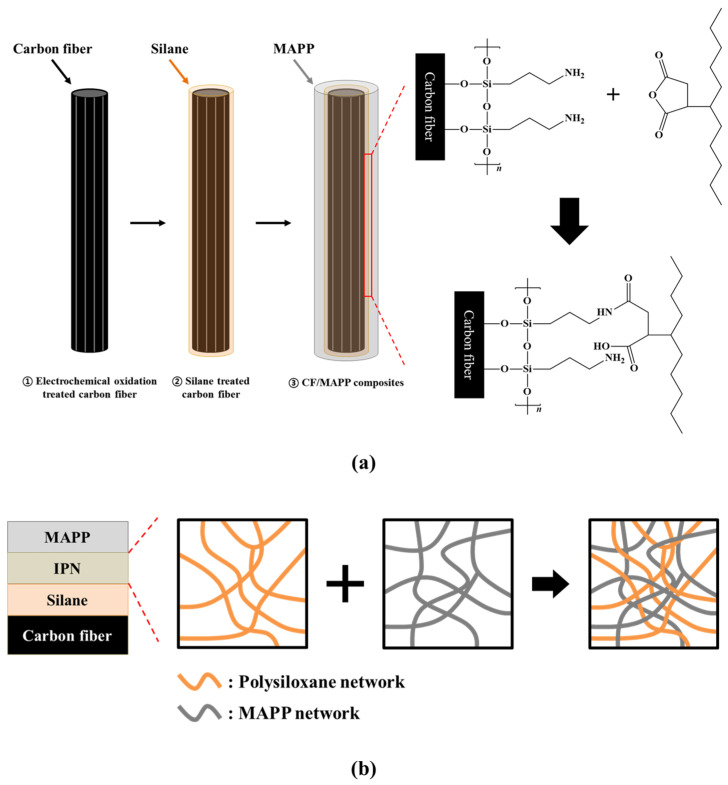
Schematic of the interfacial adhesion enhancement mechanism between the silane-treated carbon fiber and maleic anhydride-grafted polypropylene; (**a**) carbon fiber and maleic anhydride-grafted polypropylene covalent bonding mechanism, (**b**) mechanism of interpenetrating polymer network formation.

The interaction between the surface-treated carbon fiber and polymer matrix is an important factor in the mechanical properties of the fabricated CFRTPs. The physical mixing of the surface-treated carbon fibers and thermoplastic matrix can improve mutual adhesion through intermolecular entanglement or acid–base interaction. The surface-treated EO-CF, EOS1-CF, EOS2-CF, and EOS3-CF samples exhibited higher IFSS than AS-CF. The oxygen functional group formed on the carbon fiber surface after electrochemical oxidation and the -NH_2_ functional group formed on the carbon fiber surface after silane treatment were considered to affect the increase in the IFSS owing to the covalent bonding with polar MAPP. In addition, the improvement of the interfacial adhesion can be interpreted as the formation of a cage-type interpenetrating polymer network (IPN) composed of a polysiloxane network, and the amino group has a strong affinity for the hydroxyl group of the fiber. The polysiloxane network hydrogen-bonded with the hydroxyl group formed on the carbon fiber surface during silane treatment can form IPN due to the entanglement of the molecular chain of the thermoplastic matrix, improving the interfacial adhesion between the fiber and matrix [21]. The binding mechanism of the carbon fiber and MAPP according to the carbon fiber surface treatment is shown in Figure 8.

## 4. Conclusions and Future Perspectives

In this study, a two-step surface treatment comprising electrochemical oxidation and silane treatment was performed to improve the bonding strength between carbon fibers and a thermoplastic resin. In the surface analysis, the SEM and AFM images confirmed the etching of the carbon fiber surface during electrochemical oxidation, resulting in shallow longitudinal grooves and the formation of more grooves. Subsequently, the formation of a silane layer on the surface of the carbon fiber during the silane treatment was confirmed, which increased the roughness. The FTIR analysis confirmed the formation of Si-O_X_ groups on the surface of the silane-treated carbon fibers through the hydrogen bonding between the hydroxyl group introduced to the carbon fiber surface by electrochemical oxidation treatment and hydrolyzed silanol. In addition, the XPS analysis noted the increase in the N_1s_ peak owing to the influence of the siloxane network end group -NH_2_ formed on the carbon fiber surface. From the measurement of the mechanical properties, the tensile strength of the silane-treated carbon fiber increased by up to 21% (4.7 GPa) compared to the untreated carbon fiber, indicating the increase in the tensile strength by filling the grooves of the carbon fiber. IFSS also increased the strength of the silane-treated carbon fibers by up to 450% (11.8 MPa) compared to the untreated carbon fibers. The IFSS of the carbon fiber/MAPP composite was considered to improve owing to the increase in the specific surface area due to the increase in roughness after the surface treatment and covalent bonding of the -NH_2_ functional group and MAPP of the terminal group of the siloxane network formed on the surface of the carbon fiber. Consequently, the formation of covalent bonds between silane and the thermoplastic matrix substantially improved the mechanical properties of the carbon fibers and thermoplastic composites. Therefore, the proposed surface treatment facilitated continuous processing and improved the chemical activity of the carbon fiber surface to produce CFRTPs with excellent mechanical properties. The improvement of the interfacial adhesion of CFRTPs has the potential to replace metals in various vehicles, including automobiles and aircraft.

## Figures and Tables

**Table 1:** Treatment conditions and sample names.

Sample Name	Treatment Conditions
AS-CF	Unsized carbon fiber
EO-CF	Electrochemical-oxidation-treated carbon fiber
EOS1-CF	Silane-treated carbon fiber at a concentration of 1 wt.%
EOS2-CF	Silane-treated carbon fiber at a concentration of 2 wt.%
EOS3-CF	Silane-treated carbon fiber at a concentration of 3 wt.%

## Data Availability

Not applicable.
